# A New lncRNA, *APTR*, Associates with and Represses the *CDKN1A/p21* Promoter by Recruiting Polycomb Proteins

**DOI:** 10.1371/journal.pone.0095216

**Published:** 2014-04-18

**Authors:** Masamitsu Negishi, Somsakul P. Wongpalee, Sukumar Sarkar, Jonghoon Park, Kyung Yong Lee, Yoshiyuki Shibata, Brian J. Reon, Roger Abounader, Yutaka Suzuki, Sumio Sugano, Anindya Dutta

**Affiliations:** 1 Department of Biochemistry and Molecular Genetics, University of Virginia School of Medicine, Charlottesville, Virginia, United States of America; 2 Department of Microbiology, Neurology, University of Virginia, Charlottesville, Virginia, United States of America; 3 The Institute of Medical Science, The University of Tokyo, Tokyo, Japan; Virginia Commonwealth University, United States of America

## Abstract

Long noncoding RNAs (lncRNAs) have emerged as a major regulator of cell physiology, but many of which have no known function. CDKN1A/p21 is an important inhibitor of the cell-cycle, regulator of the DNA damage response and effector of the tumor suppressor p53, playing a crucial role in tumor development and prevention. In order to identify a regulator for tumor progression, we performed an siRNA screen of human lncRNAs required for cell proliferation, and identified a novel lncRNA, *APTR*, that acts in *trans* to repress the *CDKN1A/p21* promoter independent of *p53* to promote cell proliferation. *APTR* associates with the promoter of *CDKN1A/p21* and this association requires a complementary-*Alu* sequence encoded in *APTR*. A different module of *APTR* associates with and recruits the Polycomb repressive complex 2 (PRC2) to epigenetically repress the *p21* promoter. A decrease in *APTR* is necessary for the induction of p21 after heat stress and DNA damage by doxorubicin, and the levels of *APTR* and *p21* are anti-correlated in human glioblastomas. Our data identify a new regulator of the cell-cycle inhibitor *CDKN1A/p21* that acts as a proliferative factor in cancer cell lines and in glioblastomas and demonstrate that *Alu* elements present in lncRNAs can contribute to targeting regulatory lncRNAs to promoters.

## Introduction

Long noncoding RNAs (lncRNAs), transcribed from outside (intergenic) or within protein coding regions (intragenic), are >200 nucleotides in length but do not code for proteins. Thousands of lncRNAs have been identified in mammalian cells, many with expression patterns specifically restricted by cell or tissue-type and development stage [1], [2]. The few lncRNA that have been functionally characterized often regulate gene expression, both transcriptionally and post-transcriptionally [3]. LncRNA *Xist* is a *cis*-acting RNA responsible for the initiation and spreading of inactivation of the very X chromosome that it is transcribed from, by interacting with and recruiting the Polycomb repressive complex 2 (PRC2) [4] which is key epigenetic regulator during development and tumorigenesis [5], [6] and represses gene expression at the sites where they are recruited by methylating histone H3 on lysine 27. The *Xist*-PRC2 complex is recruited to the X-chromosome inactivation center by DNA binding protein YY1 [7]. A larger group of *trans*-acting lncRNAs, like *HOTAIR*, regulate gene expression at sites away from where they are transcribed, by recruiting to these sites chromatin-modifying complexes such as PRC2, LSD1 and CoREST/REST [8], [9]. It remains unclear, however, how these *trans*-acting lncRNAs are recruited to their target gene locations.

The cyclin-dependent kinase (CDK) inhibitor *p21* is expressed ubiquitously and associates with and inhibits kinases important for G1/S transition such as cyclin D/CDK4, Cyclin D/CDK6 and Cyclin E/CDK2 [10], [11]. The suppression of CDK activity allows the accumulation of hypophosphorylated Rb, which represses the E2F transcription factor to cause G1 phase cell cycle arrest [12]. p21 is a critical molecule for inhibiting cell proliferation in normal and cancer cells and is regulated at multiple levels, most notably at the transcriptional level by the tumor suppressor p53 when the latter is activated by DNA damage [13].

In this study, we identify a novel lncRNA *Alu*-mediated p21 transcriptional regulator (*APTR*) necessary for cell proliferation. *APTR* represses *p21* transcription by recruiting the PRC2 complex to the *p21* promoter. The complementary *Alu* (c-*Alu*) element embedded in *APTR* is required for the localization of *APTR* to the *p21* promoter, suggesting that embedded *Alu* elements in lncRNAs can contribute to the functions of lncRNAs. Cellular stresses that induce *p21* such as heat shock and doxorubicin treatment down-regulate *APTR*, and this downregulation is important for the induction of *p21* independent of whether p53 is active or not. A survey of gliomas suggests that *APTR* and *p21* levels are anti-correlated. Our results identify a new regulator of *p21*, an lncRNA *APTR*, that silences *p21* epigenetically by recruiting PRC2 to the *p21* promoter.

## Materials and Methods

### Ethics statement

Ten fresh frozen primary glioblastoma multiforme specimens and two normal brain tissue samples were obtained from patients undergoing surgical treatment at the University of Virginia Hospital following written informed consent and in accordance with a protocol approved by the University of Virginia's Institutional Review Board for Health Sciences Research. These tumors have been studied in two previous publications [14] [15]. All tumor specimens were prepared from patients who did not receive radiotherapy prior to surgery. The correlation coefficient plot with r and P value was generated using GraphPad Prism (GraphPad Software, Inc.).

### Plasmid construction

The full length nucleotide sequence of *APTR* was obtained from the FLJ cDNA library. For MS2 pulldown assays, plasmid pUC-MS2 coat protein (MS2BP) fused to YFP (Addgene plasmid 27122) and plasmid pUC-24MS2 were used. The 24 copies of MS2 stem loops were amplified from the 24MS2 stem loop cassette (Addgene 45162) by PCR and inserted at the 3′ end of *APTR* in the pcDNA3-*APTR* plasmid (Invitrogen). For luciferase assays, various lengths (3.7 kb, 2.7 kb and 152 bp) of the *p21* promoter with/without the *Alu*-containing region (−3389 to −2595 from the TSS) were cloned by PCR using 293T cell genomic DNA and inserted into the HindIII sites of the pGL4.20 plasmid (Promega).

### Statistical analysis

In all the figures, Student's t-test was used for testing whether the differences were statistically significant, and analyzed using GraphPad Prism (GraphPad Software, Inc.). Statistical significance was defined at P<0.05.

### Cell culture, synchronization and transfection

MCF10A, PC3, 293T and U87 cells were grown in DMEM, glioma cell line A172 and HCT116 cells were grown in McCoy's 5a Medium supplemented with 10% fetal bovine serum and penicillin/streptomycin. MCF10A, PC3, 293T, HCT116, A172 and U87 were purchased from the American Type Culture Collection (ATCC, Manassas, VA). HCT116 and p53−/−, p21−/− derivatives were supplied by Dr. B. Vogelstein [16]. For siRNA transfection, we used Lipofectamine^TM^2000 or RNAiMAX (Invitrogen) according to the manufacturer's protocols. The siRNA sequences are provided in Table S1. For flow cytometry analysis, MCF10A cells were treated with BrdU (1 µM/ml) for 30 min and fixed by 70% Ethanol at −20°C. After 1 hr fixation, fixed cells were stained by BrdU-FITC antibody (BD biosciences) for 1 hr and analyzed by FACS analysis with propidium iodide (50 µg/ml). For nocodazole treatment, asynchronous 293T cells were transfected by si*GL2*, *APTR*#1 or #2. After 24 hrs of siRNA transfection, cells were treated with nocodazole (0.1 µg/ml) for 16 hrs and then harvested and analyzed by FACScaliber (BD biosciences) for DNA content.

### Long noncoding RNA screening and MTT/BrdU incorporation assays

Three different siRNAs were designed against each of the 286 candidate lncRNAs to avoid off-target effects, and transfected into PC3 and MCF10A cells using three 96-well plates with three technical replicates. Two to three days after transfection, cells were incubated with 10 µM BrdU for 15 min to 1 hr, and fixed with FixDenat (Roche, Indianapolis, IN) for 30 min. Cells were blocked with 3% BSA in PBS for 1 hr, then incubated with HRP-coupled anti-BrdU antibody (Roche) diluted in 3% BSA in PBS for 1 hr. After washing three times with PBS containing 0.1% TX-100, cells were incubated with TMB substrate (Pierce, Rockford, IL) for 5–10 min and analyzed by the absorbance at 450 nm following the addition of 1 M H_2_SO_4_ to stop the reaction. For data analysis, each assay plate contained four wells of negative control (luciferase; *GL2*) and two wells of positive control (*ORC2*) siRNAs for normalization. To normalize values of BrdU incorporation, we calculated the inhibition index (%) using the following equation: Inhibition index of gene *X* (%)  =  (*GL2*av−*X*)/(*GL2*av−*ORC2*av) ×100, where *X*, *GL2*av, and *ORC2*av represent BrdU incorporation (absorbance at 450 nm) of gene *X* and average of *GL2* and *ORC2*, respectively. Genes with a SD of inhibition indices (from 3 technical replicates) greater than a cutoff value were eliminated to select technically reproducible data.

### RNA purification, RT-PCR and Quantitative PCR

Total RNA was extracted from cells using Trizol total RNA isolation reagent (Invitrogen). cDNA was synthesized with oligo (dT)_18_ or Random hexamer by the Superscript III First Strand Synthesis System for RT-PCR (Invitrogen) according to the manufacturer's instructions. Quantitative RT-PCR was performed by iCycler iQ™ Real time PCR Detection System (BioRad) with SYBR green Master Mix (BioRad). The Ct values were determined with the default threshold setting. Relative expressions for the target RNAs were determined by the comparative CT (2^−ΔΔCt^) method after normalization to *GAPDH*.

### *APTR* knockdown/rescue assays

After 6hrs of siRNA transfection, 293T cells were transfected by control or *APTR* deletion mutant-encoding plasmids. Total RNA was analyzed by RT-PCR or Q-RT-PCR after 72 rs of siRNA transfection. The knockdown efficiency (more than 80% reduction) of endogenous *APTR* in each experiment was confirmed by Q-RT-PCR using the primer set 1 and 2 (Table S1).

### Cell proliferation assays

For cell proliferation assays, HCT116 *p21^+/+^* and HCT116 *p21* ^−/−^ were seeded at 1×10^6^ cells/well in 6-cm plates and transfected by siRNA against *GL2*, *APTR* after 24 hours. Viable cells were counted every two days by Trypan Blue exclusion using auto cell counter (Invitrogen), and then replated at 1×10^6^ cells/well. All experiments were performed on three biological replicates. P values were calculated by two-way ANNOVA analysis using GraphPad Prism (GraphPad Software, Inc.).

### Northern blot analysis

Total RNA was extracted from 293T cells using TRIzol reagent (Invitrogen). Poly(A) RNA was purified from total RNA using a PolyA TRACT kit (Promega) according to the manufacturer's protocol. Northern blot analysis was performed by standard protocol. [α-^32^P]-dCTP-labeled DNA oligonucleotides were made by Rediprimer II DNA labeling system (GE healthcare). The probes were generated from a 300 nt fragment of *APTR* cDNA (651–950), amplified using the following primer sets:

Forward, 5′-TGTGGGTACAAAAGGAGAGTAACAT-3′;

Reverse, 5′-GTAGATCTGGAGCTGCAACTACAG-3′.

### Heat shock and Doxorubicin treatment

For heat shock, 293T cells were treated at 37, 42 or 55°C for 30 min followed by incubation at 37°C for 60 min and then total RNA was extracted. For rescuing *p21* transcription, cells were transfected with pcDNA3 or pcDNA3-*APTR*1-2303 for 48 hr and incubated at 37°C or 55°C for 30 min followed by recovery at 37°C for 60 min. HCT116 cells were treated with Doxorubicin (0, 10, 30 µM) for 16 hrs and total RNA was extracted. After cDNA synthesis, RNA expression was analyzed by RT-PCR. The primer sets are provided in Table S1.

### Immunoprecipitation analysis

Immunoblot and immunoprecipitation analysis were performed as described previously with slight modifications [17]. Histone H1 kinase assay was performed as previously [18]. The following antibodies were used: anti- α-Tubulin (B-5-1-2), Cyclin E1 (HE111 and C-19) (Santa Cruz); p21 (CP36) (Millipore); phosphor-RB (ser807/81; D20B12) (Cell signaling). Total RB (4H1) (Cell signaling).

### Crosslinking immunoprecipitation with MS2 for DNA or RNA purification

After 48 hrs of cotransfection of *APTR*-MS2 and MS2BP-YFP plasmids into 293T cells, cells were harvested. For MS2-CLIP assay of *APTR* in EZH2 or SUZ12 knockdown cells, 6 hrs following transfection of siRNAs against EZH2 or SUZ12, MS2BP-YFP and *APTR*-MS2 or MS2 plasmids were cotransfected into 293T cells and cells were harvested after 48 hours. Cells were then crosslinked with 1% Glutaraldehyde for 10 min and homogenized with the ChIP lysis buffer followed by sonication in the same way as the ChIP assay described below. The lysate was immunoprecipitated by ChIP grade anti-GFP antibody (Abcam, ab290) which can crossreact with the YFP protein. For MS2 pulldown/chromatin isolation, after washing and decrosslinking by heat incubation at 65°C overnight, DNA was extracted with phenol/chloroform after treatment with RNaseA at 37°C for 30 min followed by proteinase K at 55°C for 60 min. Samples were analyzed by qPCR, using specific primer sets provided in Table S1.

### Chromatin immunoprecipitation assay

Chromatin immunoprecipitation assays were performed as described previously with slight modifications [17]. Briefly, 293T cells were crosslinked with 1% formaldehyde for 15 min and neutralized in the presence of 0.125 M Glycine. The cells were homogenized with ChIP lysis buffer [(50 mM Tris-HCl (pH 8.0), 10 mM EDTA, 0.1%SDS, and a proteinase inhibitor cocktail (Complete Mini) (Roche). Each sample was immunoprecipitated with pre-mixed antibody-dynabead protein G complex. The following antibodies were used: ChIP grade anti-rabbit IgG (Abcam, ab46540), anti-SUZ12 and anti-H3K27me3 (Millipore, 07-449). The immunoprecipitant was incubated at 65°C overnight to decrosslink and treated with RNaseA at 37°C for 30 min and proteinase K at 55°C for 60 min followed by phenol/chloroform treatment. Purified DNA was analyzed by qPCR analysis using the primers provided in Table S1.

### RNA immunoprecipitation and biotinylated RNA pulldown assays

For RNA immunoprecipitation and biotinylated RNA pulldown, 293T cells (5×10^6^) were fixed with 1% formaldehyde and lysed in hypertonic lysis buffer [20 mM sodium phosphate at pH 7.0, 250 mM NaCl, 0.1% NP-40, 1 mM EDTA, 1 mM DDT and a protease inhibitor cocktail (Complete Midi, Roche)] followed by sonication, and then subjected to RNA immunoprecipitation by the following antibodies; anti-EZH2 (Cell Signaling, AC22), SUZ12 (Abcam, ab12073) and rabbit polyclonal IgG antibody. For biotinylated RNA pulldown, biotinylated sense/antisense wild type or deletion mutants of *APTR* were generated by MEGAscript T7 kit (Life technologies) according to the manufacturer's protocol. Whole cell extracts derived from 293T cells were pulled down by Streptavidin sepharose beads-Biotinylated *APTR* complex after 12 hr incubation at 4°C. The precipitated RNA was subject to cDNA synthesis and analyzed by RT-PCR. The primer sets are provided in Table S1.

### Dual luciferase reporter assays

293T cells were seeded on 6-well plates and co-transfected by Firefly luciferase reporter fused to various lengths of the *p21* promoter (200 ng), control Renilla luciferase reporter (5 ng) and either empty vector or vector expressing wild type or deletion mutants of *APTR* (2 ug). After 48 hrs of transfection, the lysates were analyzed by Dual-Luciferase reporter assay system (Promega) according to the manufacturer's protocol. Firefly luciferase activity of each reporter was normalized to the Renilla luciferase activity.

## Results

### A targeted siRNA screen for lncRNAs required for cell proliferation identifies *APTR*

We previously reported a targeted RNAi (TARCOR) screen for genes required for cell proliferation, where we performed a manual, moderate-throughput siRNA transfection against a targeted gene set and measured effects on cell proliferation or viability by a quantitative comparison of BrdU incorporation in wells transfected with different siRNAs [19]. We applied the same method to identify lncRNAs required for cell proliferation and viability. The ‘Full-length Long Japan’ (FLJ) collection of sequenced human full length cDNA [20] was screened for those that did not contain an open reading frame to find 286 putative lncRNAs. We designed 3 different siRNAs against each lncRNA to guard against off-target effects, and transfected them separately into MCF10A (*p53*-wild type, non-transformed breast epithelial cells). BrdU incorporation was measured in each well after 72 hrs of siRNA transfection and compared to that in wells transfected with si-*GL2* (negative control) and si-*ORC2* (a known essential factor for cell proliferation). Knockdown of 74 lncRNAs inhibited BrdU incorporation to at least 90% of the level of inhibition seen with si-*ORC2* (Table S2). Among these, we searched for lncRNAs for which there was evidence of moderate to high expression in RNA-seq data from MCF10A cells. In this paper we focus on one of these lncRNAs, *Bramy2034329*/*SLV01230*, which was renamed by us as *APTR*.

**Figure 1:**
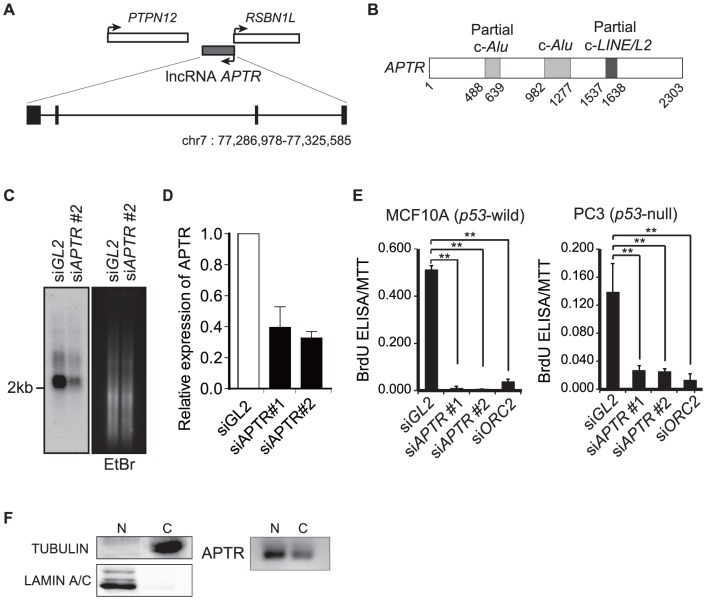
LncRNA *APTR* knockdown inhibits cell proliferation in a *p53*-independent manner. (A) Schematic of the *APTR* locus. Black boxes represent exons. (B) Schematic of lncRNA *APTR*. “c-“: complementary to *Alu* or *LINE* elements. (C) Northern blot of *APTR* on poly(A) RNA in 293T cells transfected with si*GL2* or si*APTR#2*, at 72 hr after siRNA transfection. Probe: *APTR* cDNA (651–950). (D) Relative *APTR* expression levels normalized to GAPDH in 293T cells transfected with si*GL2* or si*APTR#1*, *#2*, at 72 hr after siRNA transfection. (E) *APTR* knockdown inhibits cell proliferation. BrdU incorporation (DNA synthesis) was measured by BrdU ELISA, 72 hr after siRNA transfection, and normalized to MTT assays (number of viable cells). si*Orc2* was used as a positive control known to inhibit cell proliferation. **: P<0.005. (F) Subcellular expression levels of *APTR* in 293T cells. α-TUBULIN and LAMIN A/C were analyzed as markers for cytoplasm or nuclear fraction (left panel). *APTR* expression levels in the fractions were analyzed by RT-PCR (right panel).

*APTR* is a 2303-nucleotides lncRNA expressed from the opposite strand of the intergenic region between *PTPN12* and *RSBN1L* in chromosome 7q21 (Figure 1A, Figure S1). RepeatMasker (http://www.repeatmasker.org) found that *APTR* possesses two sequences complementary to *SINE/Alu* elements (c-*Alu*) as well as one sequence complementary to *LINE/L2* element (Figure 1B and Figure S2). Northern blot analysis on poly(A)+RNA derived from 293T cells identified a 2.3 kb RNA that is decreased by siRNA against *APTR* (Figure 1C). Consistent with our lncRNA screening results, knockdown of *APTR* by two different siRNAs not only decreased proliferation of MCF10A, but also PC3 (prostate cancer cell with mutant p53) cells to the same extent as knockdown of ORC2 (Figure 1D and E). Since *APTR* is enriched by 5′ oligo capping method (Figure S1) and polyA RNA purification (Figure 1C) [20], we conclude that *APTR* is a capped, poly(A) tailed and spliced lncRNA expressed mainly in nucleoplasm (Figure 1F) and required for proliferation of multiple cell-lines.

### *APTR* regulates cell proliferation via *p21* transcriptional suppression

**Figure 2:**
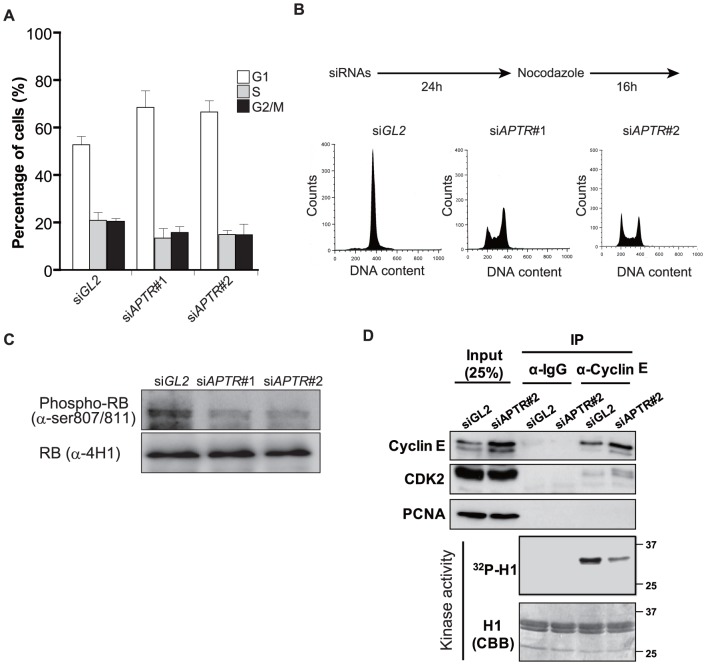
*APTR* depletion suppresses the G1/S phase progression. (A) MCF10A cells transfected with *siAPTR #1* or *#2* accumulate in the G1 phase of the cell-cycle as measured by two color FACS for propidium-iodide and BrdU (mean ±s.e.m., n = 3). (B) 293T cells transfected with siGL2 or *siAPTR #2* were analyzed by FACS for propidium-iodide in presence of Nocodazole (0.1 µg/ml). Schematic of the Nocodazole treatment procedure was presented on the top. (C) Immunoblot shows that Retinoblastoma protein is hypo-phosphorylated in 293T cells transfected with *siAPTR #1 and #2*. Total RB protein was analyzed as a loading control. (D) Reduced kinase activity of Cyclin E/CDK2 in 293T cells transfected with si*APTR*#2. Cyclin E1 and CDK2 were analyzed by IP and immunoblot with indicated antibodies. PCNA was analyzed as a loading control. Kinase activity: autoradiogram of 32P labeled histone H1 after *in vitro* kinase assays with immunoprecipitates. CBB; coomassie blue staining to show equal amounts of H1 were added to all the lanes.

To explore the molecular mechanism for cell proliferation, we next examined whether *APTR* regulates cell cycle progression. Depletion of *APTR* by two different siRNAs in asynchromous MCF10A resulted in an increased G1 population (Figure 2A). Progression of 293T cells through the cell-cycle was also analyzed after *APTR* knockdown in the presence of nocodazole, a chemical that blocks cells in M phase. After 24 hrs of si*APTR*#1 or #2 transfection, asynchronous 293T cells were treated with nocodazole (0.1 µg/ml) for 16 hrs, and then analyzed by FACS analysis for propidium iodide staining for DNA content. As shown in Figure 2B, while *siGL2* transfected control cells accumulated in M phase, depletion of *APTR* led to an increase of G1 and S populations. In agreement with the increase in G1 population, depletion of *APTR* resulted in a decreased RB phosphorylation (Figure 2C). Cyclin E1/CDK2 kinase was decreased in activity on histone H1 *in vitro* (Figure 2D). These findings suggest that *APTR* is important for G1-S transition most likely due to a requirement for the activity of CyclinE/CDK2 kinase [12].

**Figure 3:**
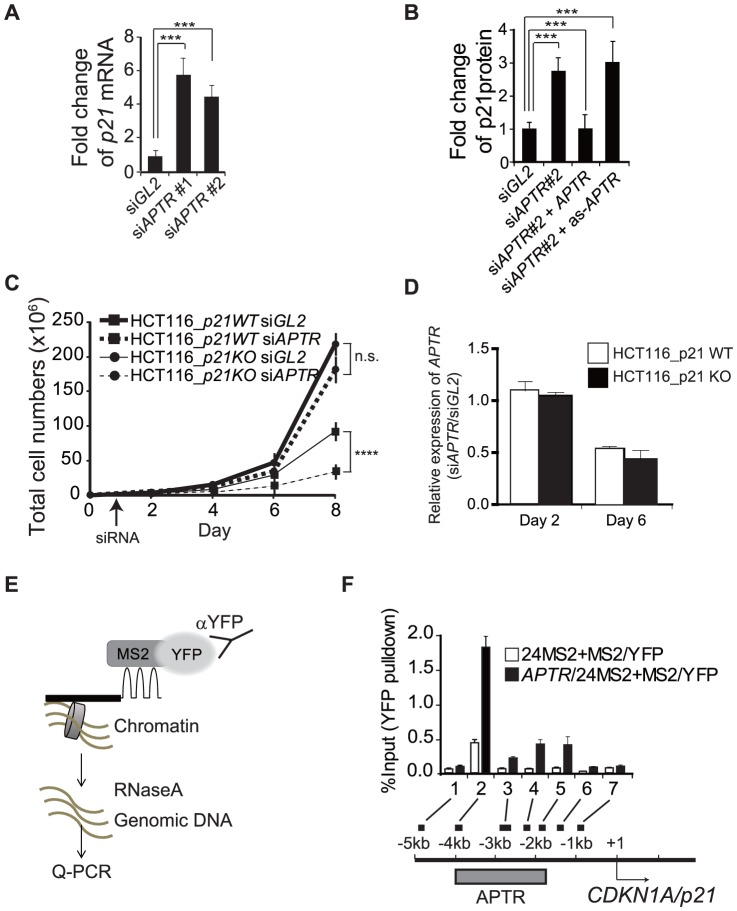
*APTR* suppresses *p21* transcription. (A) Q-RT-PCR shows induction of *p21* mRNA (normalized to *GAPDH*) after *siAPTR*. Fold change compared to *siGL2*-transfected 293T cells (mean ±s.e.m., n>6, ***: P<0.0005). (B) The induction of p21 protein in the si*APTR*#2 transfected 293T cells is prevented by overexpression of sense but not antisense *APTR*. Fold change of p21 normalized to ACTIN, compared to si*GL2*-transfected cells (mean ±s.e.m., n>3, **: P<0.005). (C) Growth suppression after *siAPTR* is alleviated in *p21* ^−/−^ HCT116 cells. Mean ±s.e.m. n = 9. Right: *APTR* expression levels (Normalized to *GAPDH*) measured by Q-RT-PCR in *p21* ^+/+^ or *p21* ^−/−^ HCT116 cells at the indicated days after transfection of siRNAs (n.s.: not significant, ****: P<0.0001). (D) Q-RT-PCR shows fold change of *APTR* (normalized to GAPDH), compared to the si*GL2*-transfected cells in the two cell lines in C (mean ±.e.m., n = 3). Note that cells were transfected on Day 1, so Day 2 is 1 day after transfection and Day 6 is 5 days after transfection. Thus si-*APTR* does not decrease *APTR* on day 1 after transfection, but the *APTR* RNA remains low up to day 5. (E) Schematic of MS2-CLIP. The dark line is *APTR* RNA fused to MS2 binding sequences. (F) *APTR* associates with the *p21* promoter. **Top**: The % of input DNA present in the MS2BP-YFP CLIP is shown in cells expressing *MS2* alone or *MS2*-*APTR* (mean ±s.e.m, n>6). 1–7 refer to the primer pairs in the schematic. **Bottom**: locations of *p21* promoter fragments amplified by primer pairs 1–7 in the CLIP assay. Grey bar: area where *APTR* binds.

Next, we surveyed the levels of CDK inhibitors in the *APTR* knockdown cell lines and found that *p21* mRNA and protein were increased after *APTR* knockdown (Figure 3A and B). Induction of p21 after *APTR* knockdown can be prevented by over-expression of exogenous sense-strand *APTR* (but not anti-sense *APTR*) (Figure 3B), suggesting that induction of p21 is specifically due to *APTR* knockdown. The exogenous *APTR* is expressed at a sufficiently high level that enough *APTR* remains in the cells even after si*APTR* transfection (Figure S3). Notably, depletion of *APTR* induced p21 even in *p53*-deficient cell lines (Figure S4), indicating that *APTR* suppresses p21 in a *p53*-independent manner.

Is p21 induction necessary for inhibition of cell proliferation upon *APTR* knockdown? To address this, we observed cell growth in *p21* ^−/−^ HCT116 cell lines [21] transfected with si*APTR*. Knockout and siRNA-mediated knockdown of *p21* attenuated the cell growth inhibition upon *APTR* knockdown (Figure 3C, 3D and Figure S5), suggesting that the cell growth inhibition in si*APTR* transfected HCT116 cells was dependent on p21 induction. We next sought to determine whether *APTR* is physically associated with the *p21* promoter. *MS2* tagging of RNA is widely used for following RNA localization in eukaryotic cells [22], [23]. The 24 repeats of MS2-binding stem loops of bacteriophage *MS2* RNA fused to *APTR* (*APTR/MS2*) is bound by bacteriophage MS2 binding protein (MS2BP) fused to fluorescent protein YFP (MS2BP/YFP). We adapted the *MS2* tagging system for MS2-crosslinking and immunoprecipitation (MS2-CLIP) to enrich the chromatin bound to *MS2* tagged RNA (Figure 3E). Optimization experiments revealed that 1% glutaraldehyde fixation is needed to obtain reproducible results upon chromatin immunoprecipitation using anti-YFP antibody (data not shown). MS2-crosslinking and immunoprecipitation (MS2-CLIP) demonstrated that *APTR* binds to multiple DNA sites between −1.6 and −4 kb relative to the transcription start site (TSS) of *p21* (Accession: NM_000389) (Figure 3F), indicating that *APTR* may suppress the *p21* promoter directly.

### *APTR* interacts physically with the Polycomb repressive complex2

**Figure 4:**
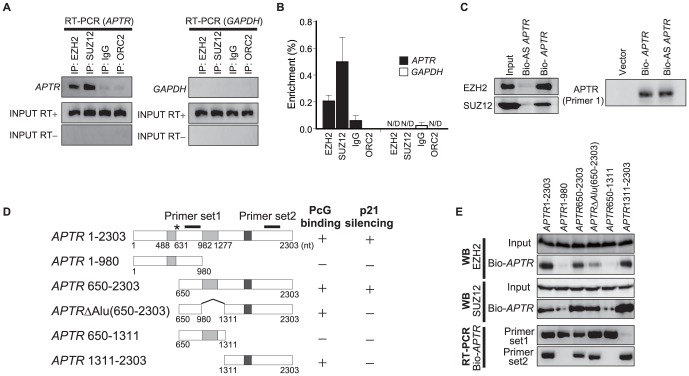
*APTR* interacts with PRC2 proteins EZH2 and SUZ12. (A) *APTR* interacts specifically with EZH2 and SUZ12 *in vivo*. **Top**: RNA Immunoprecipitates of 293T cell lysates probed for *APTR* by RT-PCR using primer set 1 in Fig. 4D. *GAPDH* was analyzed as a negative control in the right. IgG, ORC2: IP negative controls. **Middle and bottom**: input RNA analyzed with and without RT. (B) Enrichment of endogenous *APTR* in the RNA immunoprecipitates of endogenous EZH2 and SUZ12. RNA immunoprecipitation analysis in Figure 4A was analyzed by Q-RT-PCR. IgG and ORC2 were analyzed as a negative IP control. *GAPDH* was analyzed as a negative control. N/D represents not detectable. Error bars indicate Mean ±s.e.m. (n = 3). (C) **Left**: Immunoblot for EZH2 and SUZ12 after mixing *in vitro* transcribed biotinylated sense-/antisense-*APTR* with cell lysates and pull-down on streptavidin beads. **Right**: RT-PCR to show equal levels of biotinylated sense- or antisense-*APTR* (input RNA). (D) Schema of wt *APTR* (as in Fig. 1B) and deletion mutants with locations of primer sets 1 and 2 used to detect *APTR*. Summary of Figure 4D (PcG binding) and Figure 6A (*p21* silencing) indicated on the right. (E) EZH2 and SUZ12 interact with the 3′-portion of *APTR* in an experiment similar to Figure 4C. Top 4 panels: Proteins input or pulled down by biotinylated *APTR* detected by immunoblots. Bottom 2 panels: WT and mutant *APTR* were pulled down at comparable levels on strepavidin beads as detected by RT-PCR with primer sets 1 and 2 (note that some deletions can be detected only by one primer pair).

Recent studies revealed that the repressive H3K27 trimethylation mark is regulated by PRC2 on the *p21* promoter and is responsible for stable *p21* gene silencing [24], [25]. PRC2 contains EZH2, the enzyme that catalyzes the repressive trimethylation of histone H3 on lysine 27 (H3K27me3) [6] and SUZ12, a cofactor required for the catalytic activity of EZH2 [26]. To address the molecular mechanism by which *APTR* silences the *p21* gene, we examined the interaction between *APTR* and the PRC2 complex in RNA immunoprecipitation assays with antibodies to EZH2 and SUZ12 followed by RT-PCR (Figure 4A) and Q-RT-PCR (Figure 4B). *APTR* is present in immunoprecipitates of EZH2 or SUZ12, but not of ORC2 (negative control protein). An unrelated RNA, *GAPDH* mRNA was also not precipitated with EZH2 or SUZ12 (Figure 4A, right panel). Conversely, *in vitro* transcribed biotinylated sense *APTR* associated *in vitro* with cellular EZH2 and SUZ12 and pulled them down on streptavidin beads (Figure 4C), but antisense *APTR* did not do so. Biotinylated *APTR* with specific deletions (Figure 4D and E) showed that the 3′ end of *APTR* (1311–2303) lacking any *Alu* elements was sufficient for binding EZH2 and SUZ12, but portions containing only the c-*Alu* sequences (1–980 or 650–1311) could not bind EZH2 and SUZ12 (Figure 4E). These findings indicate that the 3′ portion of *APTR* specifically interacts with the PRC2 complex.

**Figure 5:**
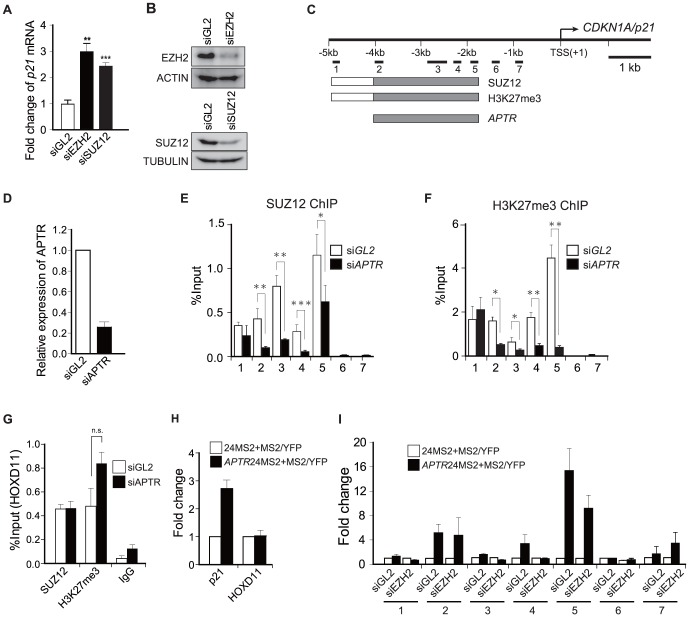
*APTR* regulates *p21* epigenetically via the PRC2 complex. (A) Q-RT-PCR shows induction of *p21* mRNA normalized to *GAPDH* after *EZH2* or *SUZ12* knockdown on the left. Fold change compared to si*GL2*-transfected cells. Mean ±s.e.m, n>6, **: P = 0.006. ***: P<0.0001). (B) Endogenous EZH2 and SUZ12 were analyzed by immunoblot after EZH2 or SUZ12 knockdown as a control for Figure 5A. (C) Schematic of *p21* promoter and primer pairs for CLIP PCR (as in Figure 3F). Boxes: binding of indicated proteins/RNA in control cells (Figure 3F, 5E–F). Grey areas: regions where binding is downregulated by *siAPTR#2*. (D) qRT-PCR shows *APTR* knockdown efficiency by *siAPTR#2* in Figure 5E–G. (E–F) ChIP of SUZ12 or H3K27me3 on the *p21* promoter in 293T cells transfected by either *siGL2* or *siAPTR#2*. X-axes: primer-pairs 1–7 in Figure 3F. Y-axes: %Input values were presented. Mean ±s.e.m. n = 6. (G) ChIP of SUZ12 or H3K27me3 on the *HOXD11* locus in 293T cells transfected by either *siGL2* or *siAPTR#2*. X- axis: antibodies used for ChIP. Y-axis: % input of *HOXD11* locus in precipitates. (n.s.: not significant, Mean ±s.e.m. n = 6) (H) CLIP of MS2-*APTR* or *MS2* RNA alone in the *p21* promoter (primer-pair 2 in Figure 3F) and the *HOXD11* locus. Mean ±s.e.m. n = 6. Y-axis shows amount of specific DNA in the precipitate normalized to that in the CLIP of *MS2* alone. (I) CLIP of *MS2*-*APTR* or *MS2* RNA alone on the *p21* promoter in 293T cells transfected by si*GL2* or si*EZH2*. X-axes: primer-pairs 1-7 in Figure 3F. Y-axis: amount of specific DNA in the precipitate normalized to that in CLIP of *MS2* alone. Mean ±s.e.m. n = 3.

We next examined whether the PRC2 complex is recruited to the *p21* promoter by *APTR* and whether its catalytic activity is required for the repression of the *p21* gene. Depletion of *EZH2* or *SUZ12* by siRNA induces *p21* mRNA in 293T cells (Figure 5A and B). The ChIP of endogenous SUZ12 revealed that SUZ12 is localized at −1.6 to −4.7 kb relative to the TSS of *p21* (si*GL2* in Figure 5E; summarized as boxes labeled SUZ12 in Figure 5C). These sites overlap with the −1.6 to −4 kb region where *APTR* binds to the *p21* promoter (*APTR* box, Figure 5C and Figure 3F). Knockdown of *APTR* significantly decreased the recruitment of SUZ12 to the −1.6 to −4 kb region of the *p21* promoter (si*APTR* in Figure 5E; grey parts of the SUZ12 boxes in Figure 5C).

Notably, the −1.6 to −4.7 kb area of the *p21* promoter was also enriched for H3K27me3 (Figure 5F; H3K27me3 box in Figure 5C), and knockdown of *APTR* selectively decreased the repressive H3K27me3 modification at −1.6 to −4 kb relative to the TSS (Figure 5F; grey part of the H3K27me3 box in Figure 5C). The global level of H3K27me3 does not change after *APTR* knockdown (Figure S6).

The ChIP enrichment of SUZ12 and H3K27me3 in the *p21* promoter are comparable to that seen at the known PRC2 target locus, *HOXD11* [27] (si*GL2* in Figure 5G). Knockdown of *APTR* did not decrease SUZ12 or H3K27me3 at the *HOXD11* locus (si*APTR* in Figure 5G). Compatible with these results, MS2-CLIP showed no enrichment of *APTR* in the *HOXD11* locus (Figure 5H). Thus *APTR* is co-localized with the PRC2 complex selectively at the *p21* locus and not at all PRC2-bound promoters. The increase of H3K27me3 at the HOXD11 promoter after knockdown of *APTR* is clearly not due to an increase in recruitment of PRC2 (SUZ12 in Fig. 5G), and not due to an increase in global levels of H3K27me3 (Fig. S6). The increase could be due to a local decrease in demethylation, which may not be directly dependent on *APTR*, but is beyond the scope of this paper.

Next, we asked whether the PRC2 complex is required for recruitment of *APTR* to the *p21* promoter. Fig. 3F shows that *APTR* is maximally recruited to sites 2, 4 and 5 in the *p21* promoter. MS2-CLIP of *APTR* after siGL2 and siEZH2, showed that *APTR* was still recruited to sites 2 and 5 after EZH2 depletion (Figure 5I), though there was a slight decrease at site 4 and increase in site 7. The results suggest that PRC2 is not required to recruit *APTR* to two of the three major sites of recruitment in the *p21* promoter.

Collectively, these results indicate that the PRC2 complex is recruited by *APTR* to the *p21* promoter specifically between −1.6 to −4 kb relative to the TSS, where the complex catalyzes the repressive modification of chromatin to repress the *p21* promoter.

### Identification of the portions of *APTR* required for *p21* gene silencing

**Figure 6:**
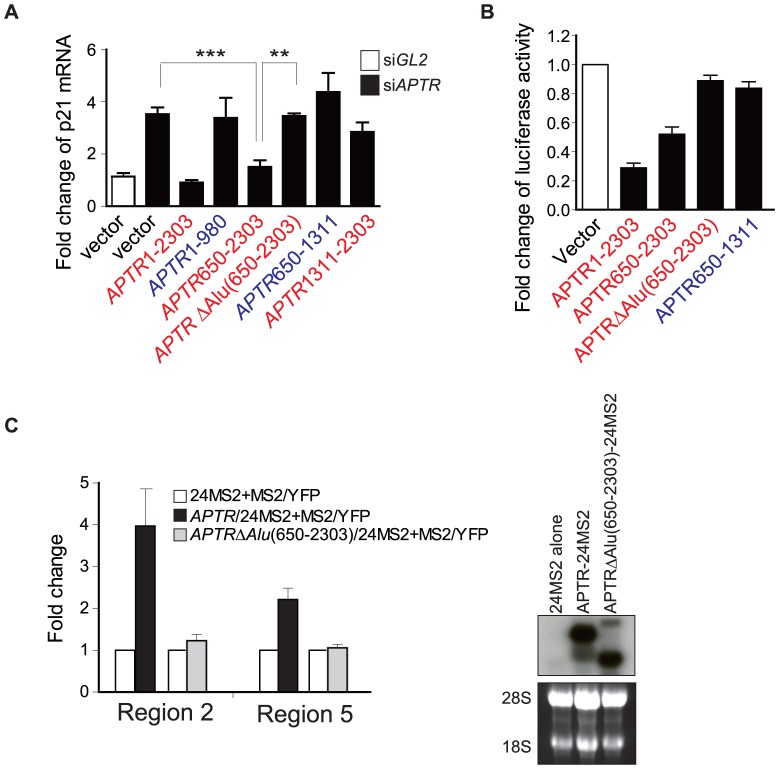
The embedded c-*Alu* and the 3′ end of *APTR* are each required for *p21* suppression. (A) Q-RT-PCR of *p21* mRNA after *siAPTR#2* (black bars) and transfection of empty vector or vector expressing wild-type or mutant *APTR* in 293T cells. X-axis: empty vector or form of *APTR* expressed by transfected plasmid. Red represents the mutants that bind to PRC2. Blue represents the ones that do not bind to PRC2. Y-axis: fold change of *p21* normalized to *GAPDH* relative to *siGL2*-transfected cells receiving empty vector (white bar). Mean ±s.e.m; n = 6, ***: P<0.0005. **: P<0.005. (B) Firefly luciferase activity of *p21* promoter in cells transfected with empty vector or vector expressing wild-type or deletion mutants of *APTR*. Red represents the mutants that bind to PRC2. Blue represents the ones that do not bind to PRC2. Firefly luciferase activities were normalized to Renilla luciferase from co-transfected plasmid. (C) CLIP of *MS2-APTR* or *MS2-APTRΔAlu*(650–2303) or *MS2* RNA alone (negative control) on two sites in the *p21* promoter (2 and 5 as defined in Figure 3F). Y-axis: amount of specific DNA in the precipitate normalized to that in CLIP of *MS2* RNA alone. Mean ±s.e.m., n = 6. Northern blot on the right with *APTR* cDNA (651–950), shows that the *APTR* fusion RNAs are expressed.

To perform structure-function studies on *APTR*, we rescued the *APTR* knockdown by overexpressing exogenous *APTR* (wt or deletion mutants). Knockdown of endogenous *APTR* increases p21, but this was reversed by over-expressing exogenous full length *APTR* (Figure 6A, first three bars). Overexpression of deletion mutants of *APTR* indicated which portions of *APTR* are required for *p21* silencing (Figure 6A and summarized in Figure 4D). All the versions of exogenous *APTR* are expressed at comparable levels (Figure S7). *APTR* (650–2303), deleted of the first partial c-*Alu* element but retaining the second complete *c-Alu*, silenced *p21*. *APTR* that cannot bind PRC2 (1–980 or 650–1311) was unable to silence *p21*, suggesting that interaction with PRC2 is essential for silencing *p21*. Interestingly, *APTRΔAlu*(650–2303) missing all c-*Alu* elements did not silence the *p21* promoter even though it could bind to PRC2 (Figure 6A and Figure 4D).

We confirmed these results by co-transfecting a firefly luciferase reporter driven by the *p21* promoter (2.7 kb in length from TSS) with a plasmid expressing wt or mutant *APTR* (Figure 6B). Here again, *APTR*(650–2303) repressed the *p21* promoter, but deletions that removed the *c-Alu* elements or the 3′ end of *APTR* abrogate this repression. These findings suggest that the 3′ terminal portion and the embedded second c-*Alu* of *APTR* are both required for *p21* silencing.

**Figure 7:**
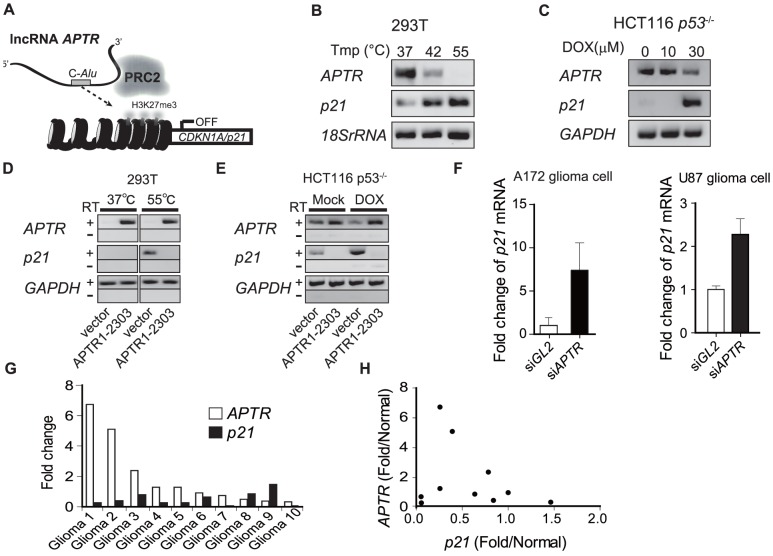
The implication of *APTR*-mediated *p21* silencing in normal cell function and cancer. (A) A schematic of the lncRNA *APTR*-mediated *p21* gene silencing. *APTR* suppresses *p21* gene expression by guiding the PRC2 complex to the *p21* promoter. (B–C) RT-PCR of indicated transcripts in indicated cells. *GAPDH* and *18SrRNA* are loading controls. (D–E) Overexpression of exogenous *APTR* (indicated at bottom) suppresses the induction of *p21* after heat shock or after Doxorubicin in indicated cells. RT: reverse transcriptase. Fewer PCR cycles were done compared to Figure 7B explaining why endogenous *APTR* is not seen. (F) *siAPTR* induces *p21* in human glioma cells. Q-RT-PCR of *p21* mRNA normalized to *GAPDH*. Mean ±s.e.m; n = 3. (G) Levels of *APTR* and *p21* mRNAs in ten GBMs. Fold change in the GBM relative to normal brain tissue (average of two normal brains). (H) Scatter plot of the data in Figure 7G to show the anti-correlation between *APTR* and *p21* RNAs. Pearson R = −0.254. unpaired t test P = 0.004, n = 10.

The 3′ portion of *APTR* is required for *APTR* to interact with PRC2. To explore whether the embedded second c-*Alu* element is required for targeting *APTR* to the *p21* promoter, we assessed the localization of *APTRΔAlu*(650–2303) in MS2-CLIP assay. As shown in Figure 6C, *APTRΔAlu*(650–2303)/MS2 was not recruited to the *p21* promoter. Consistent with this and Figure 6B, over-expressing exogenous *APTR/MS2* suppressed the p21 promoter luciferase activity (2.7kb in length from TSS), but *APTRΔAlu(650*–*2303)/MS2* failed to do so (data not shown). These findings suggest that the c-*Alu* element of *APTR* is important for recruiting the *APTR*-PRC2 complex to the *p21* promoter (schematic in Figure 7A).

### Decrease of *APTR* correlates with *p21* induction by cellular stress and in glioblastomas

Finally, we examined whether the regulation of *p21* by *APTR* is important for normal cellular physiology or for disease. Cellular stresses that induce p21, such as heat shock (Figure 7B), or the DNA damage-inducing chemotherapy drug doxorubicin (Figure 7C), down-regulate *APTR* expression while up-regulating *p21*. Over-expression of exogenous *APTR* prevents induction of *p21* after heat shock or doxorubicin-induced DNA damage (Figure 7D and 7E), indicating that the decrease of *APTR* is important for the induction of *p21* following heat shock or DNA damage.

Glioblastoma multiforme (GBM), the most aggressive primary brain cancer in humans, often inactivates the *p53*–*p21* signaling pathway [28], [29]. p21 is also critical for the radiation sensitivity and/or chemosensitivity of GBM cells [30], [31]. We wondered whether variation in *APTR* expression could affect *p21* expression in human GBM. siRNA depletion of *APTR* in two GBM cells (A172 and U87) up-regulated *p21* mRNA (Figure 7F) and caused cell growth inhibition (Figure S8). Notably, co-knockdown of *p21* in the U87 cells alleviates the growth inhibition produced by APTR knockdown (Figure S8). In addition, quantitative RT-PCR of total RNAs derived from 12 frozen GBM samples showed anti-correlation of *APTR* and *p21* mRNAs (Pearson r = −0.2549, P = 0.0396) in these GBM samples (Figure 7G, 7H and Figure S9). Therefore the level of expression of *APTR* is important for determining the expression of *p21* in GBM cell lines and tumors.

## Discussion

### A novel lncRNA *APTR* is a bona fide *p21* regulator for cell proliferation

We identify a new lncRNA, *APTR*, which is expressed from a distant site in the genome, but targets the *p21* promoter *in trans* for epigenetic repression. Depletion of *APTR* resulted in the dissociation of the PRC2 complex throughout the *APTR* localization sites in the *p21* promoter (Figure 3 and 5). Most interesting, cellular stresses that induce *p21*, also decrease the expression level of the repressor *APTR* and forced expression of *APTR* inhibited p21 induction in the stressed condition (Figure 7B–E). Numerous studies show that *p21* gene is regulated at multiple levels, transcriptional, post-transcriptional and post-translational, in response to intra- or extracellular stress [13]. We identified an lncRNA regulator that plays an important role in the constitutive suppression of p21 and in the induction of p21 by stress, independent of *p53*.

Despite the number of independent factors that regulate p21, it is heartening to see that *APTR* and *p21* levels were anti-correlated in glioblastomas, suggesting that at least in these tumors *APTR* is a significant repressor of *p21*. We do not know whether the variation in levels of *APTR* stem from genetic differences between the tumors or differences in the growth rate, oxygenation or other factors that affect the level of stress on the tumors. In addition, larger, well-annotated tumor sets will be examined in the future to ascertain whether *APTR* levels increase with glioma progression and/or whether *APTR* is an useful prognostic marker both for survival and for response of the tumor to radio- or chemotherapy.

The *p21* gene locus expresses several *cis*- and *trans*-acting lncRNAs in normal and stressed cells. lncRNA *linc-p21* and *PANDA* are respectively transcribed from ∼15 or 5 kb upstream to the *p21* TSS, induced by p53 upon DNA damage and involved in the regulation *in trans* of multiple genes downstream of p53 [32], [33]. However, the transcriptional regulation of *p21* itself is independent of *linc-p21* and *PANDA*, [32], [33]. In contrast, antisense-p21 (*AS-p21*) epigenetically suppresses *p21* transcription in *cis*, when transcribed from the antisense strand and overlapping with the *p21* transcript [34]. *APTR* is clearly very different from these known lncRNAs, in that it is encoded elsewhere in the genome, and acts *in trans* on the *p21* promoter itself.

### Interaction of *APTR* with PRC2 is necessary for p21 silencing

One essential activity of *APTR* is its ability to interact with PRC2 so that the latter can be recruited to the *p21* promoter. More than 20% of lncRNAs are bound by PRC2 in various cells [8], [9], and some of these lncRNAs, like *XIST* and *HOTAIR*, interact with local chromatin or DNA binding proteins to recruit the chromatin modifying machinery to specific sites in the target loci [1], [4], [7]. *APTR* similarly appears to recruit PRC2 to the *p21* promoter, although not to all PRC2 target loci, as exemplified by *HOXD11*. Future studies will explore which proteins in PRC2 interact with *APTR* and what sequence or structural features of *APTR* are essential for interaction with PRC2. Clearly significant structural studies are necessary to understand exactly how these critical protein:RNA interactions are established and what determines the specificity and affinity of the various PRC2:lncRNA interactions. An interesting question is whether different lncRNAs that interact with PRC2 have similar or different affinities for the protein complex and whether they compete with each other to regulate gene expression by targeting PRC2 to certain sites in the genome and away from other sites. It will also be interesting to know in the future whether certain lncRNAs alter the conformation and enzymatic activity of PRC2 complex, or whether their activity is limited to the targeting of PRC2 to specific sites in the genome.

### The embedded complementary *Alu* element in *APTR* is required to recruit the lncRNA to the *p21* promoter

Importantly, the c-*Alu* deletion mutant of *APTR* could not suppress *p21* transcription because the mutation prevented *APTR* recruitment to the *p21* promoter. We do not know how *APTR* interacts with the *p21* promoter and how the *APTR*-embedded *Alu* element is required for this interaction. For example, is there a specific “receptor” for the c-*Alu* element, or *c-Alu*-bound proteins, in the *p21* promoter? Or does the *c-Alu* region of *APTR* play a structural role so that *APTR* can fold in the correct conformation before it can interact with the relevant targeting proteins? Regardless of the mechanism this is another example of how *Alu* elements encoded in RNAs are functionally important.

Interestingly, the *APTR* localization sites in the *p21* promoter (−1.6 kb to −4 from the TSS) contains tandem inverted partial and full *Alu* elements (Figure S10). Furthermore, we observed at least two uncharacterized promoter lncRNAs transcribed across the *Alu* element from both strands of this site at very low levels (our unpublished data). Thus, we tested the possibility that *Alu*: c-*Alu* base-pairing interactions between *cis*-lncRNAs from the *p21* promoter and *APTR* tether the latter to the *p21* promoter. Luciferase assays on *p21* promoter with or without the *Alu* region (3.7 kb or 2.7 kb of the promoter upstream from the TSS, respectively), however, clearly showed that *APTR* suppressed the *p21* promoter without the *Alu* region (Figure S10). Thus a simple *Alu*:*c-Alu* base-pairing interaction between the *p21* promoter and *APTR* is not the explanation for how *APTR* is recruited to the promoter.

An alternative possibility is that RNA binding proteins interact with the c-*Alu* sequence of *APTR* to recruit the latter to the *p21* promoter. Past studies on *SINE/Alu* revealed that some proteins bind physically to *Alu* DNA or RNA [35], [36]. Examples include signal recognition particle SRP9/14 heterodimer interacting with *Alu* element containing RNAs in the cytoplasm [37] and double strand RNA binding protein Staufen 1 (STAU1) recognizing intermolecular *Alu*: c-*Alu* element pairs and flanking sequences [38]. Thus another possibility is that similar factor(s) bind to the *c-Alu* element in *APTR* to target it to the *p21* promoter.

Clearly much more needs to be done to understand what decides target specificity of *APTR*. Genome-wide ChIP-seq studies of *APTR*-binding sites on the genome and proteomic studies of proteins that bind to *APTR* will be helpful in this regard. However, our data raise the intriguing possibility that embedded repetitive elements in an lncRNA could contribute to the targeting of an lncRNA to the genome.

## Supporting Information

Figure S1 **The nucleotide sequence of long noncoding RNA** ***APTR*** **.** Shaded boxes represent sequences in the RNA complementary to *SINE/Alu* elements and open box represents sequence in the RNA complementary to *LINE/L2* elements.
(EPS)

Figure S2 **Alignment of sequences from the repetitive elements in** ***APTR*** **with the retrotransposon** ***SINE/Alu*** **or** ***LINE/L2*** **.**
(EPS)

Figure S3 **Enough overexpressed** ***APTR*** **persists after si** ***APTR*** **transfection to exceed the amount of endogenous** ***APTR*** **present normally.** *APTR* expression levels normalized to *GAPDH* in cells transfected either si*GL2* or si*APTR*#2 as analyzed by Q-RT-PCR. Residual expression level of the siRNA-sensitive *APTR* after knockdown is higher than that in wild type cells transfected with si*GL2* and empty vector. Percentages represent extent of reduction of *APTR* in si*APTR*-transfected cells compared to that in si*GL2*-transfected cells.
(EPS)

Figure S4 ***APTR*** **knockdown results in induction of p21 in multiple cancer cells without wild type p53.** (A) RT-PCR shows *APTR* knockdown efficiency by si*APTR*#2 in figure S4B. (B) Induction of p21 protein in indicated cells transfected with si*GL2* or si*APTR*#2. PC3 and H1299 have mutant, and 293T has inactivated p53.
(EPS)

Figure S5 **Knockdown of p21 alleviates cell growth inhibition upon** ***APTR*** **knockdown.** (A) Growth suppression after *siAPTR* is alleviated in *p21* knockdown HCT116 cells. The indicated siRNAs were transfected at Day1. Data represents total cell number at Day 0 and Day 4 (after 3 days of transfection) with mean ±s.e.m., n = 3, *:P<0.05. (B) The expression levels of *APTR* and *p21* relative to si*GL2*-transfected cells were measured by Q-RT-PCR at Day 6 (after 5 days of transfection).
(EPS)

Figure S6 Expression level of Histone H3K27me3 does not change after *APTR* knockdown. Immunoblot of H3K27me3 in 293T cells transfected with si*GL2* or si*APTR*#2.
(EPS)

Figure S7 **Expression of exogenous APTR RNA in** ***APTR*** **knockdown-** ***APTR*** **rescue assays.** (A) All cells express the expected exogenous *APTR* RNA in Figure 6A. RT-PCR with primer sets shown in Figure 4C. RT: reverse transcriptase. (B) RNA expression change of the siRNA-sensitive *APTR* WT and mutants (1–980) after transfection of si*APTR*#2. Percentages represent reduction rate of *APTR* in si*APTR*-transfected cells compared to that in si*GL2*-transfected cells. The residual level of *APTR1*-980 after *siAPTR* is still significantly higher than endogenous *APTR* levels.
(EPS)

Figure S8 **Knockdown of p21 alleviates cell growth inhibition in** ***APTR*** **knockdown glioma cells.** (A) MTT assays (after 48 hrs of siRNA transfection) show that growth suppression after si*APTR* is alleviated in *p21* knockdown U87 glioma cells. Data represents mean ±s.e.m., n = 3, **:P<0.005. (B) Q-RT-PCR shows that the expression levels of *APTR* and *p21* relative to si*GL2*-transfected cells in Fig. S8A.
(EPS)

Figure S9 **Anti-correlation between** ***APTR*** **and** ***p21*** **RNAs in human glioma cells.** The two technical replicates of the experiment in Figure 7G.
(EPS)

Figure S10 **The tandem inverted** ***Alu*** **elements in the** ***APTR*** **regulatory p21 promoter is not involved in the** ***APTR*** **-mediating** ***p21*** **silencing.** (A) Schema represents firefly luciferase plasmid construction with various length of *p21* promoter. (B) Firefly luciferase activity of various *p21* promoters in cells transfected with empty vector or vector expressing wild-type *APTR*. Firefly luciferase activities were normalized to Renilla luciferase from co-transfected plasmid (mean±s.e.m; n = 3).
(EPS)

Table S1 **Sequences of primers and siRNAs used for RT-PCR, Q-RT-PCR, ChIP/CLIP assays and siRNA interference in this study.**
(EPS)

Table S2 **siRNA based lncRNA functional screening in MCF10A cells.** F-BRAMY2034329_1 was renamed as *APTR*. The positive control si*ORC2* and negative control si*GL2* were transfected in six to eight wells in each biological replicate. The inhibition of BrdU incorporation by each siRNA is expressed as a % of the extent of inhibition by si*ORC2*. For each lncRNA, we showed the % inhibition for each of three siRNAs and the average and standard deviation of the inhibition. Each siRNA was tested in triplicate. The average inhibition was calculated from all three siRNAs, unless the SD>0.2 x mean, in which case the outlier siRNA was discarded when calculating the average.
(XLSX)

Method S1 **Supplementary Method.**
(DOC)
