# Supplementary material for: A New lncRNA, APTR, Associates with and Represses the CDKN1A/p21 Promoter by Recruiting Polycomb Proteins
Source: PLoS One. 2014 Apr 18;9(4):e95216. doi: 10.1371/journal.pone.0095216 (PMC3991591; doi:10.1371/journal.pone.0095216)
Supplement: Method S1 — Supplementary Method. (DOC) [file pone.0095216.s013.doc]

Supplementary methods

**A new lncRNA, *APTR,* associates with and represses the *CDKN1A/p21* promoter by recruiting Polycomb proteins**

Masamitsu Negishi^1^, Somsakul P. Wongpalee^1^, Sukumar Sarkar^1^, Jonghoon Park^1^, Kyung Yong Lee^1^, Yoshiyuki Shibata^1^, Brian J. Reon^1^, Roger Abounader^2^, Yutaka Suzuki^3^, Sumio Sugano^3^ and Anindya Dutta^1^

**Materials and methods**

**Cell proliferation assays**

For cell proliferation assays, cells (HCT116 cell line) were seeded at 0.5×10^6^ cells/well in 6-cm plates (Day 0) and transfected by siRNA against *GL2*, *APTR and p21* after 24 hours (Day 1). Viable cells were counted every two days by Trypan Blue exclusion using auto cell counter (Invitrogen), and then replated at 1.0×10^6^ cells/well. All experiments were performed on three biological replicates. P values were calculated by Student’s t-test analysis using GraphPad Prism (GraphPad Software, Inc.). The siRNA sequences are provided in Table S1.

**MTT assays**

MTT [3-(4,5-dimethyl-2-thiazolyl)-2,5-diphenyl-2*H*-tetrazolium bromide] assays were performed according to the manufacturer's instruction (Promega, CellTiter96 nonradioactive cell proliferation assay). Briefly, cells (U87 glioma cell line) were seeded at 0.2x10^4^ cells/well in 24 well plates and were transfected by siRNA against *GL2*, *APTR and p21* next day. After 24 hrs of siRNA transfection, cells were transferred to 96 well plates and were treated with MTT for 4 hrs and then measured the fluorescence (after 48 hrs of siRNA transfection). The experiments were performed on three biological replicates. The siRNA sequences are provided in Table S1.
